# Supplementary material for: Time-series transcriptome analysis identified differentially expressed genes in broiler chicken infected with mixed Eimeria species
Source: Front Genet. 2022 Aug 8;13:886781. doi: 10.3389/fgene.2022.886781 (PMC9393255; doi:10.3389/fgene.2022.886781)
Supplement: Supplementary file 2 [file DataSheet1.ZIP › 4dpi_KEGG.Gsea.1625100298584/KEGG_HISTIDINE_METABOLISM.html]

Details for gene set KEGG\_HISTIDINE\_METABOLISM[GSEA]

|  || Dataset | TMM\_4dpi\_gct\_format\_4dpi\_gct\_format.Class\_4dpi.cls #PC\_versus\_NC.Class\_4dpi.cls #PC\_versus\_NC\_repos |
| Phenotype | Class\_4dpi.cls#PC\_versus\_NC\_repos |
| Upregulated in class | 1 |
| GeneSet | KEGG\_HISTIDINE\_METABOLISM |
| Enrichment Score (ES) | 0.54109126 |
| Normalized Enrichment Score (NES) | 1.5641587 |
| Nominal p-value | 0.025914093 |
| FDR q-value | 0.2725114 |
| FWER p-Value | 0.8828 |
Table: GSEA Results Summary

  

Fig 1: Enrichment plot: KEGG\_HISTIDINE\_METABOLISM      
 Profile of the Running ES Score & Positions of GeneSet Members on the Rank Ordered List

  

| SYMBOL | TITLE | RANK IN GENE LIST | RANK METRIC SCORE | RUNNING ES | CORE ENRICHMENT || 1 | AOC1 | na | 69 | 1.800 | 0.1563 | Yes |
| 2 | CNDP1 | na | 269 | 1.215 | 0.2491 | Yes |
| 3 | HEMK1 | na | 442 | 1.022 | 0.3269 | Yes |
| 4 | ALDH1A3 | na | 581 | 0.895 | 0.3959 | Yes |
| 5 | FTCD | na | 858 | 0.731 | 0.4388 | Yes |
| 6 | ASPA | na | 967 | 0.685 | 0.4914 | Yes |
| 7 | DDC | na | 1072 | 0.648 | 0.5411 | Yes |
| 8 | MAOB | na | 3877 | 0.180 | 0.3234 | No |
| 9 | HDC | na | 4345 | 0.138 | 0.2969 | No |
| 10 | ALDH3A2 | na | 5110 | 0.069 | 0.2394 | No |
| 11 | MAOA | na | 5750 | 0.013 | 0.1873 | No |
| 12 | METTL2B | na | 6336 | -0.033 | 0.1415 | No |
| 13 | TRMT11 | na | 7216 | -0.110 | 0.0781 | No |
| 14 | HNMT | na | 7475 | -0.133 | 0.0686 | No |
| 15 | HAL | na | 8692 | -0.250 | -0.0104 | No |
| 16 | LCMT2 | na | 9771 | -0.382 | -0.0659 | No |
| 17 | AMDHD1 | na | 9987 | -0.412 | -0.0467 | No |
| 18 | ALDH3B2 | na | 10162 | -0.439 | -0.0217 | No |
| 19 | ALDH7A1 | na | 10220 | -0.448 | 0.0138 | No |
| 20 | LCMT1 | na | 10251 | -0.453 | 0.0521 | No |
| 21 | ALDH9A1 | na | 10288 | -0.458 | 0.0903 | No |
| 22 | METTL6 | na | 10938 | -0.591 | 0.0894 | No |
Table: GSEA details [plain text format]

  

Fig 2: KEGG\_HISTIDINE\_METABOLISM      
 Blue-Pink O' Gram in the Space of the Analyzed GeneSet

  

Fig 3: KEGG\_HISTIDINE\_METABOLISM: Random ES distribution      
 Gene set null distribution of ES for **KEGG\_HISTIDINE\_METABOLISM**
